# Benign breast disease and breast cancer risk in African women: A case-control study

**DOI:** 10.21203/rs.3.rs-3301977/v1

**Published:** 2023-09-01

**Authors:** Olasubomi J. Omoleye, Jincong Q. Freeman, Mojisola Oluwasanu, Adenike Adeniji-Sofoluwe, Anna E. Woodard, Benjamin S. Aribisala, Prisca O. Adejumo, Atara Ntekim, Timothy Makumbi, Paul Ndom, IkeOluwapo O. Ajayi, Olufunmilayo I. Olopade, Dezheng Huo

**Affiliations:** University of Chicago; University of Chicago; University of Ibadan; University of Ibadan; University of Chicago; Lagos State University; University of Ibadan; University of Ibadan; Mulago Hospital; Hôpital Général Yaoundé; University of Ibadan; University of Chicago; University of Chicago

**Keywords:** Benign breast disease, Breast cancer, Risk factors, Africa, Nigeria

## Abstract

**Purpose:**

To examine the association between benign breast disease (BBD) and breast cancer (BC) in a heterogeneous population of African women.

**Methods:**

BC cases and matched controls were enrolled in three sub-Saharan African countries, Nigeria Cameroon, and Uganda, between 1998–2018. Multivariable logistic regression was used to test the association between BBD and BC. Risk factors dually associated with BBD and BC were selected. Using a parametric mediation analysis model, we assessed if selected BC risk factors were mediated by BBD.

**Results:**

Of 6418 participants, 55.7% (3572) were breast cancer cases. 360 (5.7%) self-reported BBD. Fibroadenoma (46.8%) was the most reported BBD. Women with a self-reported history of BBD had greater odds of developing BC than those without (adjusted odds ratio [aOR] = 1.47, 95% CI: 1.13–1.91). Biopsy-confirmed BBD was associated with BC (aOR = 3.11, 95% CI: 1.78–5.44). BBD did not significantly mediate the effects of any of the selected BC risk factors.

**Conclusions:**

In this study, BBD was associated with BC and did not significantly mediate the effects of selected BC risk factors.

## Introduction

Breast cancer is the second leading cause of cancer-related deaths worldwide, with a lifetime risk of 1 in 23 among Eastern African women and about 1 in 46 among Western African women [1, 2]. The association between Benign Breast Disease (BBD) and breast cancer is documented in the literature, and the magnitude of the relationship varies by BBD lesion type [3–6]. While the consensus is that BBDs are not precursor lesions for breast cancer, research has suggested that BBDs may indicate a background proliferative state of the breast that could herald a cancerous process [3, 7], with some studies indicating that the presence of BBD might sometimes reflect a hyperestrogenic state [4, 8].

Studies examining the relationship between BBD and breast cancer have mainly been conducted in non-African populations [16]. Current literature on BBD in Africa hitherto has been merely descriptive, mostly hospital-based, and performed on relatively small datasets [9–17]. Aside from a published abstract [1], there is no published large-scale study assessing the association between BBD and breast cancer in African women, nor are there published studies evaluating BBD as a mediator of other breast cancer risk factors. Given the high prevalence of BBD in young women and the rising burden of premenopausal breast cancer on the African continent [25], we sought to understand the contribution of BBD to breast cancer risk in African women.

Given the paucity of data on BBD and breast cancer risk in African women, and to address this crucial gap in the literature, we conducted this study aiming to a) examine the relationships between established breast cancer risk factors and BBD, b) assess the association between BBD and breast cancer; and c) evaluate the mediating effect of BBD on the associations between breast cancer and selected risk factors, in an ethnically diverse dataset of women in Nigeria, Uganda, and Cameroon.

## Materials and Methods

### Study design & population

The Nigerian Breast Cancer Study (NBCS) started at Ibadan, Oyo State, Nigeria, in March 1998 and, using the same protocol and data collection instruments was subsequently expanded to include centers in Lagos, Nigeria, Yaoundé, Cameroon, and Kampala, Uganda in 2011, to become the African Breast Cancer Study (ABCS). The study protocol was approved by the institutional review boards in all centers. The study setting and design in the various sites have been previously described [20–24].

At The University College Hospital (UCH) in Ibadan, cases were recruited at or soon after their clinic visits following clinical confirmation of invasive breast cancer. Controls were recruited from several randomly selected communities within the UCH catchment area in Ibadan. We also recruited hospital controls from general outpatient and ophthalmology clinics in UCH. Lagos State Teaching Hospital (LASUTH) served primarily for case recruitment. At Mulago Hospital in Uganda, cases were recruited through the Department of Surgery’s Breast and Endocrine Unit; controls were randomly selected from the general outpatient clinics and surgical wards and matched to cases on age and ethnicity. At Yaoundé General Hospital in Yaoundé, cases were enrolled through the Department of Oncology, and controls, matched by age and ethnicity, were recruited from general medicine and obstetrics and gynecology departments. By the end of 2018, 6438 patients were enrolled in ABCS. After excluding males (n = 14) and patients with considerable amounts of missing risk factor data (n = 6), 6418 remained and were analyzed in this study.

### Data collection and measurements

After providing written informed consent, participants completed a structured interviewer-administered questionnaire that collected information on demographics, anthropometrics (i.e., height, waist-hip ratio, and body mass index [BMI]), history of BBD, family history of breast cancer, menstrual and reproductive history (i.e., age at menarche, age at thelarche, age at first live birth, duration of breastfeeding), physical activity, past medical history, and hormonal contraceptive use. History of BBD was self-reported, physician-diagnosed, and/or biopsy-confirmed. In the current analysis, the history of BBD was considered positive if it was diagnosed at least a year before breast cancer diagnosis (for cases) or study recruitment (for controls) to keep the correct time sequence for causal interpretation. Histologic types of BBD were also enquired, including fibroadenoma, breast cysts, intraductal papilloma, and atypical hyperplasia, as selectable options, and an open-ended option for other histologic types. Inflammatory conditions such as mastitis and breast abscesses were not considered positive BBD history for this study. We also collected data on whether BBD was biopsy-confirmed, age at BBD, date of BBD diagnosis, breast side of BBD, and whether surgical resection was performed.

### Statistical analysis

Demographic characteristics were compared between cases and controls using chi-square or Fisher’s exact tests for categorical variables and Student’s t or Wilcoxon rank-sum tests for continuous variables. Logistic regression examined the relationships between BBD and demographics, anthropometric measurements, and breast cancer risk factors. Three multivariable logistic regression models were fit to assess the association between BBD and breast cancer. Model 1 included demographics (age, level of education, study country, ethnicity, and menopausal status). Model 2 included reproductive factors (total duration of breastfeeding, parity, the age at first live birth) and variables in Model 1. Model 3 included all covariates in Model 2, anthropometries (height, waist-hip ratio), and family history of breast cancer. Both crude odds ratio (OR) and adjusted OR (aOR), with 95% confidence intervals (95% CI), were calculated.

We evaluated BBD as a possible mediator of breast cancer and selected risk factors which were based on dual association with breast cancer and BBD in our sample and the existing literature. We followed recommendations of the AGReMA statement in reporting mediation analysis results [27]. Alcohol intake, family history of breast cancer, nulliparity or low parity, late menopause, higher adolescent growth velocity, and taller height have been associated with an increased risk of both BBD and breast cancer [26–29]. However, obesity and oral hormonal contraceptives, risk factors for breast cancer, are associated with a reduced risk of BBD [7, 27, 30, 31]. Based on significant associations found in our analysis and published literature on risk factors related to an increased risk of BBD as well as breast cancer [5, 6, 10,18, 21–23, 30, 35], we selected age at menarche, parity, age at first live birth, mean duration of breastfeeding per live birth, hormonal contraceptive use, BMI, alcohol use, and family history of breast cancer as risk factors for breast cancer which BBD could mediate. Causal mediation analysis was performed using parametric regression methods developed by VanderWeele and Vansteelandt [42, 43] and implemented in the module *paramed* in STATA [41]. We estimated the natural direct effect (NDE), the effect of selected risk factors on breast cancer risk that is *not* through BBD, and the natural indirect effect (NIE), the effect of selected risk factors on breast cancer risk that is through BBD. The total effect is the product of NDE and NIE for case-control studies. From NDE and NIE on the OR scale, we estimated the proportion mediated (PM) using the Eq. (1) below:

$$PM=\frac{NDE\;*\;\left(NIE\;-\;1\right)}{\left(NDE\;*\;NIE\;-\;1\right)}$$

## Results

### Patient characteristics

Table 1 shows the characteristics of study participants. Of 6,418 participants, 55.7% were breast cancer cases. Most (83.5%) of the women were from Nigeria, 8.9% from Cameroon, and 7.6% from Uganda. Overall, the mean age at the enrollment/interview was 46 years (SD = 12.7), and 61.2% were premenopausal. The mean age at breast cancer diagnosis was 48 years (SD = 12.0). Compared to controls, cases were more likely to have a known family history of breast cancer, be postmenopausal, have a waist-hip ratio of > 0.85, and have consumed alcohol at least once a week for ≥ 1 year, however, were less likely to have used hormonal contraceptives, were 2cm taller and had about 0.3 kg/m^2^ lower BMI on average. The duration of breastfeeding (total and mean per live birth) and age at menarche were also significantly associated with breast cancer (Table 1). See **Supplementary Table 1** for the study participant description by country and the distribution of ethnic groups.

### BBD and associated characteristics

Of 360 (5.7%) women who self-reported BBD at least a year before breast cancer diagnosis (cases) or study recruitment (controls), 27.2% reported having biopsy for BBD, 15.6% did not have biopsy done, and 57.2% had unknown/missing biopsy status. Fibroadenoma was the most common subtype in Nigeria (54.5%), while in Cameroon and Uganda, breast cysts were mostly reported (52.8% and 41.2%, respectively). Overall, 30.7% of women did not know the BBD subtype. The median age at BBD diagnosis was 31 years (IQR: 25–40) and the median interval from BBD diagnosis to breast cancer diagnosis/interview was nine years (IQR: 3–19) (Table 2).

Characteristics of study participants with and without self-reported BBD (Table 3) were compared in the control group. After adjusting for age group and menopausal status among controls, younger age at menarche, older age at first live birth, shorter duration of breastfeeding, positive family history of breast cancer, and a waist-hip ratio of ≤ 0.85 were significantly associated with higher odds of BBD. Higher education was also associated with a positive history of BBD. Higher-order parity (≥ 4) was associated with lower odds of BBD in adjusted analysis. There was no clear relationship between the odds of BBD and reported adult height.

### Association between BBD and breast cancer

Overall, breast cancer was associated with higher odds of a positive history of BBD (crude OR 1.43, 95% CI: 1.14–1.78). After adjusting for multiple covariates (model 3 in Table 4), the association between BBD and breast cancer remained statistically significant (aOR 1.47, 95% CI: 1.13–1.91). The positive association existed in all three countries, although it was only tend towards significant in Nigeria. Overall, biopsied BBD was statistically significantly associated with increased odds of breast cancer in the multivariable analysis, and the strength of association was stronger than that for self-reported BBD (aOR 3.11, 95% CI 1.78–5.44) (Table 4).

### Evaluating BBD as a mediator of selected risk factors

We identified breast cancer risk factors that were significantly associated with BBD in controls. Age at menarche, age at first live birth, mean duration of breastfeeding, parity, and family history of breast cancer were significantly associated with BBD in controls (Table 3) and are known breast cancer risk factors in the literature with associations confirmed in our study (Table 1). Waist-hip-ratio was also associated with BBD but had an opposite relationship with breast cancer in our study, thus was not included in causal mediation analysis. Although BMI, alcohol consumption, and hormone contraceptive use were not significantly associated with self-reported BBD in our dataset (Table 3), they were selected for mediation analysis because of their association with BBD according to existing literature [5, 6, 10, 21]. None of the selected breast cancer risk factors were significantly mediated through BBD (Table 5), and BBD accounted for less than 10% of the association of any one of the selected risk factors with breast cancer.

## Discussion

In this large multi-site case-control study of breast cancer in Africa, we report the association between BBD and breast cancer and examine whether BBD could be a mediator of the effects of selected risk factors on breast cancer. We observed that 6.6% of cases had physician-diagnosed BBD as compared with 4.7% in healthy controls. The median interval between BBD and breast cancer diagnosis was 9 years. We found that self-reported BBD was associated with a 47% increased odds of breast cancer and biopsy-confirmed BBD was associated with a 211% increased odds of breast cancer. The association was significant in all study countries except in Nigeria, where it was marginally significant.

Among controls, lower waist-hip ratio, younger age at menarche, higher age at first live birth, and a family history of breast cancer were associated with higher odds of BBD after adjusting for age and menopausal status in our study; this is in agreement with the existing literature on BBD-predisposing risk factors [5, 18, 21,28]. Previous studies have not shown a clear relationship between breastfeeding practices and BBD [7, 18]. However, we found that a longer (both total and mean-per-live-birth) duration of breastfeeding was inversely associated with BBD. We found that a higher level of education was positively associated with BBD, similar to findings by Dorjgochoo et al. [13], but this may be due to detection bias since more educated women are more likely to consult a physician for breast conditions and/or examine their breasts more frequently [18].

In the present study, we observed a positive association between breast cancer and self-reported physician-diagnosed (aOR = 1.47) and biopsy-confirmed BBD (aOR = 3.11). This finding is consistent with previous studies which have shown varying degrees of breast cancer risk associated with histologic subtypes of BBD [16, 38]. A systematic review and meta-analysis by Dyrstad et al. found that non-proliferative BBD, proliferative BBD without atypia, and atypical hyperplasia were associated with 1.17, 1.76, and 3.93 times increased risk of future breast cancer; BBD without specified histology was associated with 2.01 times increased risk of future breast cancer [16]. Most existing large-scale studies on BBD and breast cancer risk were performed in North America, Europe, and Asia, and we did not find any large-scale studies examining BBD as a breast cancer risk factor in African women resident in Africa [16, 38]. A study of 1,406 African American women failed to show an association between “proliferative BBD without atypia” and subsequent breast cancer risk, although “proliferative BBD with atypia” conferred over three times greater risk of subsequent breast cancer compared to women with non-proliferative lesions [12]. Another study in a multi-ethnic cohort of 4,970 women (1,341 African Americans) showed that women with proliferative BBD were 1.7 and 3.8 times (with and without atypia respectively) at greater risk for breast cancer [44].

To our knowledge, our study is the first to assess the mediating effects of BBD on breast cancer risk in a heterogeneous population of African women. Our analysis suggested that BBD did not significantly mediate any selected breast cancer risk factors although it may account for about 10% of the association between age at menarche and breast cancer and 8% between mean breast-feeding duration and breast cancer. Although we did not find any published studies assessing BBD as a mediator of breast cancer risk, a published study examining the mediating effect of mammographic density (MD) on breast cancer risk found that about 17% of the risk conferred by BBD was mediated by MD [37].

Our study is not without limitations. First, the relatively low frequency of reported non-inflammatory BBD may indicate that BBDs were underreported and thus probably underestimated. This could be partly because women in the African countries are not routinely screened for breast cancer, and as such, majorly palpable breast disease will present to the physician for evaluation. Education is significantly associated with health-seeking behavior in African women [39]. With about 39% of our study population at or below elementary education, a sizeable proportion of BBD may have gone unreported because a physician did not evaluate them. Further, delay of diagnosis and misdiagnosis is a massive problem in resource-limited settings, increasing the possibility that self-reported BBD may have represented misdiagnosis. Our observation of a stronger association of biopsied BBD than the association of any BBD suggests that misreporting might dilute a true association. Secondly, since we did not extract data on specific histologic types of BBD but instead relied on participant recall, we could not tease out proliferative vs. non-proliferate, simple vs. complex, and typical vs. atypical BBD lesions, each of which has varying degrees of association with breast cancer [16, 38]. In developing countries, it is common for physicians to offer tentative histologic diagnoses based on clinical presentations and epidemiologic patterns because of the limited access to pathological diagnostics [19]. As self-reported histologic subtypes could not be entirely relied on for accuracy, we did not analyze the association of the reported BBD subtypes with breast cancer to avoid misleading results. Lastly, participants of this study might not be representative of all women in the three African countries and other women in Africa, limiting the generalizability of our findings.

In conclusion, our findings highlight that BBD, especially when biopsy-confirmed, is an important risk factor for breast cancer and that BBD does not appear to mediate the effects of traditional breast cancer risk factors in African women. Assessing the differences in breast cancer risk for distinct histologic subtypes is worth further investigation in this population. The effects of uni- vs. multi-focal lesions and surgical treatment for BBD need also to be examined in future studies. Nonetheless, our findings suggest that when women in these regions present with BBD, they are at an elevated risk for breast cancer and should be considered in breast cancer risk assessment. Future studies using modern imaging technologies and molecular pathology are needed to confirm our findings and assess the contributions of distinct histologic subtypes of BBD to breast cancer risk.

## Figures and Tables

**Table 1:** Characteristics of study participants in the African Breast Cancer Study by case-control status

	Total (N = 6,418) n (%)	Case (n = 3,572) n (%)	Control (n = 2,846) n (%)	*P* value
**Study site**				
Nigeria	5359 (83.5)	3022 (84.6)	2337 (82.1)	0.028
Cameroon	570 (8.9)	298 (8.3)	272 (9.6)	
Uganda	489 (7.6)	252 (7.1)	237 (8.3)	
**Age** ^a^ **(years)**				
<35	1181 (18.4)	399 (11.2)	782 (27.5)	<0.001
35–44	1882 (29.3)	1056 (29.6)	826 (29.0)	
45–54	1740 (27.1)	1091 (30.6)	649 (22.8)	
55–64	1048 (16.3)	642 (18.0)	406 (14.3)	
≥65	566 (8.8)	383 (10.7)	183 (6.4)	
*Mean (SD)*	46.0 (12.7)	48.3 (12.0)	43.2 (12.9)	<0.001
**Level of education**				
Elementary or less	2441 (38.5)	1401 (39.4)	1040 (37.3)	<0.001
Secondary	1720 (27.1)	963 (27.1)	757 (27.2)	
Tertiary	1581 (24.9)	904 (25.4)	677 (24.3)	
Vocational or technical	600 (9.5)	287 (8.1)	313 (11.2)	
**BMI** ^b^				
Underweight	302 (5.0)	173 (5.2)	129 (4.7)	0.356
Normal weight	2371 (38.9)	1317 (39.4)	1054 (38.2)	
Overweight	1992 (32.7)	1091 (32.7)	901 (32.7)	
Obese	1435 (23.5)	760 (22.8)	675 (24.5)	
*Mean (SD) in kg/m^2^*	26.4 (5.5)	26.2 (5.4)	26.5 (5.6)	0.045
**Height (cm)**				
<156	1437 (23.2)	662 (19.4)	775 (27.9)	<0.001
156–160	1673 (27.0)	833 (24.4)	840 (30.2)	
161–165	1628 (26.3)	986 (28.9)	642 (23.1)	
>165	1458 (23.5)	935 (27.4)	523 (18.8)	
*Mean (SD)*	160.7 (7.3)	161.6 (7.5)	159.6 (7.0)	<0.001
**Waist-hip ratio**				
≤0.85	2727 (42.5)	1396 (39.1)	1331 (46.8)	<0.001
>0.85	3691 (57.5)	2176 (60.9)	1515 (53.2)	
*Mean (SD)*	0.86 (0.08)	0.86 (0.08)	0.85 (0.08)	<0.001
**Alcohol consumption** ^c^				
Yes	672 (10.9)	447 (12.9)	225 (8.3)	<0.001
5501 (89.1)	3029 (87.1)	2472 (91.7)	15.1 (2.2)	
**Age at menarche (years)**, mean (SD)	15.1 (2.1)	15.1 (2.0)		0.425
**Age at thelarche (years)**, mean (SD)	13.2 (1.9)	13.0 (1.8)	13.6 (2.0)	<0.001
**Age at first live birth (years)**, mean (SD)	23.1 (5.0)	23.4 (5.2)	22.8 (4.8)	<0.001
**Parity**				
0	472 (7.6)	229 (6.7)	243 (8.8)	0.002
1	563 (9.1)	297 (8.6)	266 (9.6)	
2–3	1750 (28.2)	1009 (29.3)	741 (26.8)	
3424 (55.2)	1905 (55.4)	1519 (54.9)		
**Average duration of breastfeeding per live birth (months)**				
0–6	431 (7.5)	263 (8.2)	168 (6.6)	<0.001
7–12	1826 (31.7)	1133 (35.2)	693 (27.3)	
13–18	2654 (46.1)	1427 (44.3)	1227 (48.3)	
≥18	846 (14.7)	395 (12.3)	451 (17.8)	
*Mean (SD)*	14.4 (5.7)	13.9 (5.5)	15.0 (5.8)	<0.001
**Total duration of breastfeeding (years)**				
1374 (24.0)	795 (24.9)	579 (22.9)	0.001	621 (24.6)
3–4	1475 (25.8)	854 (26.7)		
5–6	1193 (20.9)	678 (21.2)		515 (20.4)
1680 (29.4)	867 (27.1)	813 (32.2)		
**Use of hormonal contraceptives** ^d^				
Yes	1816 (29.5)	926 (26.9)	890 (32.7)	<0.001
No	4349 (70.5)	2520 (73.1)	1829 (67.3)	
**Menopausal status**				
Premenopausal	3925 (61.2)	1935 (54.2)	1990 (70.0)	<0.001
Postmenopausal	2486 (38.8)	1633 (45.8)	853 (30.0)	
**Family history of breast cancer**				
Yes	319 (5.2)	201 (5.9)	118 (4.4)	0.006
No	5771 (94.8)	3187 (94.1)	2584 (95.6)	

Abbreviations: SD, standard deviation; IQR, interquartile range; BMI, body mass index.

a The age at the time of diagnosis for cases, and at the time of interview for controls.

b Underweight BMI: <18.5 kg/m^2^; normal weight BMI: 18.5-<25 kg/m^2^; overweight BMI: 25.0-<30.0 kg/m^2^; and obese BMI: ≥30 kg/m^2^.

c At least one alcoholic beverage a week for one year or longer.

d Hormonal contraceptives include oral, injectable, implant, and intrauterine contraceptive devices.

**Table 2:** Distribution of benign breast disease by case-control status and by study country

		Total (n = 6,274) n (%)	Case (n = 3,487) n (%)	Control (n = 2,787) n (%)
All study sites				
	**History of BBD**			
	Yes	360 (5.7)	229 (6.6)	131 (4.7)
	No	5914 (94.3)	3258 (93.4)	2656 (95.3)
	**Biopsied BBD**			
	Yes	118 (2.4)	92 (3.3)	26 (1.3)
	No	4726 (97.6)	2721 (96.7)	2005 (98.7)
	**Type of BBD**			
	Fibroadenoma	108 (46.8)	81 (56.3)	27 (31.0)
	Breast cyst	50 (21.7)	23 (16.0)	27 (31.0)
	Atypical hyperplasia	2 (0.9)	2 (1.4)	0
	Other/Unknown	71 (30.7)	38 (26.4)	33 (37.9)
	**BBD interval**^a^, median (IQR)	9 (3–19)	9 (3–20)	9 (4–17)
	**Age at BBD**, median (IQR)	31 (25–40)	31 (25–41)	30 (23–39)
**Nigeria**				
	**History of BBD**			
	Yes	284 (5.4)	172 (5.8)	112 (4.9)
	No	4982 (94.6)	2804 (94.2)	2178 (95.1)
	**Biopsied BBD**			
	Yes	59 (1.5)	38 (1.7)	21 (1.4)
	No	3769 (98.5)	2246 (98.3)	1523 (98.6)
	**Type of BBD**			
	Fibroadenoma	97 (54.5)	74 (68.5)	23 (32.9)
	Breast cyst	24 (13.5)	9 (8.3)	15 (21.4)
	Other/Unknown	57 (32.0)	25 (23.2)	32 (45.7)
	**BBD interval**^a^, median (IQR)	10 (4–20)	11 (4–22)	7.5 (3–16)
	**Age at BBD**, median (IQR)	30 (23–38)	30 (24–38)	29 (23–38)
**Cameroon**				
	**History of BBD**			
	Yes	46 (8.4)	33 (11.8)	13 (4.8)
	No	504 (91.6)	247 (88.2)	257 (95.2)
	**Biopsied BBD**			
	Yes	32 (5.8)	30 (10.2)	2 (0.8)
	No	524 (94.2)	265 (89.8)	259 (99.2)
	**Type of BBD**			
	Fibroadenoma	8 (22.2)	5 (20.8)	3 (25.0)
	Breast cyst	19 (52.8)	11 (45.8)	8 (66.7)
	Atypical hyperplasia	1 (2.8)	1 (4.2)	0
	Other/Unknown	8 (22.2)	7 (29.2)	1 (8.3)
	**BBD interval**^a^, median (IQR)	6 (1–13)	2 (1–7)	9 (7–21)
	**Age at BBD**, median (IQR)	38.5 (28–44)	39 (29–44)	35 (25–43)
**Uganda**				
	**History of BBD**			
	Yes	30 (6.6)	24 (10.4)	6 (2.6)
	No	428 (93.5)	207 (89.6)	221 (97.4)
	**Biopsied BBD**			
	Yes	27 (5.9)	24 (10.3)	3 (1.3)
	No	433 (94.1)	210 (89.7)	223 (98.7)
	**Type of BBD**			
	Fibroadenoma	3 (17.7)	2 (16.7)	1 (20.0)
	Breast cyst	7 (41.2)	3 (25.0)	4 (80.0)
	Atypical hyperplasia	1 (5.9)	1 (8.3)	0
	Other/Unknown	6 (35.3)	6 (50.0)	0
	**BBD interval**^a^, median (IQR)	5 (2–15)	4 (1.5–14.5)	21 (2–30)
	**Age at BBD**, median (IQR)	37 (28–42)	38 (27.5–49.5)	32 (28–32)

Abbreviations: BBD, benign breast disease; IQR, interquartile range.

a BBD interval: the years between the date of BBD diagnosis and the date of breast cancer diagnosis for cases; and between the date of BBD diagnosis and the date of interview for controls.

**Table 3:** Distribution of risk factors for self-reported physician-diagnosed BBD among controls in the African Breast Cancer Study

	Have BBD (n = 131) n (%)	No BBD (n = 2656) n (%)	Odds ratios (95% CI)	
			Unadjusted	Adjusted^e^
Study site				
Nigeria	112 (85.5)	2178 (82.0)	1.0 (reference)	1.0 (reference)
Cameroon	13 (9.9)	257 (9.7)	0.98 (0.55–1.77)	0.94 (0.52–1.71)
Uganda	6 (4.6)	221 (8.3)	0.53 (0.23–1.21)	0.50 (0.22–1.16)
**Age group** ^a^ **(years)**				
<35	33 (25.2)	739 (27.8)	1.0 (reference)	1.0 (reference)
35–44	41 (31.3)	765 (28.8)	1.20 (0.75–1.92)	1.20 (0.75–1.92)
45–54	32 (24.4)	600 (22.6)	1.19 (0.73–1.97)	1.24 (0.71–2.17)
55–64	21 (16.0)	375 (14.1)	1.25 (0.72–2.20)	1.37 (0.63–2.98)
≥65	4 (3.1)	177 (6.7)	0.51 (0.18–1.45)	0.56 (0.17–1.87)
**Level of education**				
Elementary or less	27 (20.6)	986 (38.0)	1.0 (reference)	1.0 (reference)
Secondary	37 (28.2)	709 (27.3)	**1.91 (1.15–3.16)** *	**1.99 (1.18–3.34)** **
Tertiary	48 (36.6)	618 (23.8)	**2.83 (1.75–4.59)** ***	**3.02 (1.84–4.98)** ***
Vocational or technical	19 (14.5)	284 (10.9)	**2.44 (1.34–4.46)** **	**2.44 (1.33–4.48)** **
**BMI category** ^b^				
Underweight	8 (6.3)	119 (4.6)	1.32 (0.61–2.86)	1.40 (0.65–3.04)
Normal weight	50 (39.4)	985 (38.2)	1.0 (reference)	1.0 (reference)
Overweight	37 (29.1)	845 (32.8)	0.86 (0.56–1.33)	0.85 (0.55–1.32)
Obese	32 (25.2)	631 (24.5)	1.00 (0.63–1.57)	0.96 (0.60–1.54)
**Height category (cm)**				
<156	33 (26.0)	722 (27.8)	1.0 (reference)	1.0 (reference)
156–160	50 (39.4)	775 (29.8)	1.41 (0.90–2.22)	1.39 (0.89–2.19)
161–165	27 (21.2)	606 (23.3)	0.97 (0.58–1.64)	0.94 (0.56–1.59)
>165	17 (13.4)	498 (19.2)	0.75 (0.41–1.36)	0.72 (0.40–1.31)
**Waist-hip ratio category**				
≤0.85	75 (57.3)	1221 (46.0)	**1.57 (1.10–2.24)** *	**1.59 (1.11–2.29)** *
>0.85	56 (42.8)	1435 (54.0)	1.0 (reference)	1.0 (reference)
**Alcohol consumption** ^c^				
Yes	11 (8.8)	206 (8.2)	1.08 (0.57–2.05)	1.05 (0.56–2.00)
No	114 (91.2)	2314 (91.8)	1.0 (reference)	1.0 (reference)
**Age at menarche (years)**, mean (SD)	14.6 (2.0)	15.1 (2.2)	**0.89 (0.81–0.97)** **	**0.88 (0.81–0.97)** **
**Age at thelarche (years)**, mean (SD)	13.1 (2.1)	13.6 (2.0)	0.89 (0.78–1.02)	0.90 (0.78–1.03)
**Age at first live birth (years)**, mean (SD)	24.1 (4.6)	22.8 (4.8)	**1.05 (1.02–1.09)** **	**1.06 (1.02–1.10)** **
**Parity**				
0	13 (10.2)	227 (8.8)	1.0 (reference)	1.0 (reference)
1	17 (13.3)	246 (9.5)	1.21 (0.57–2.54)	1.09 (0.51–2.31)
2–3	39 (30.5)	691 (26.7)	0.99 (0.52–1.88)	0.75 (0.37–1.50)
≥4	59 (46.1)	1421 (55.0)	0.73 (0.39–1.34)	**0.48 (0.23–0.98)** *
**Average duration of breastfeeding per live birth (months)**				
0–6	14 (12.2)	146 (6.2)	1.0 (reference)	1.0 (reference)
7–12	44 (38.3)	636 (26.8)	0.72 (0.39–1.35)	0.71 (0.38–1.33)
13–18	45 (39.1)	1163 (49.1)	**0.40 (0.21–0.75)** **	**0.40 (0.21–0.74)** **
≥18	12 (10.4)	426 (18.0)	**0.29 (0.13–0.65)** **	**0.29 (0.13–0.65)** **
**Total duration of breast feeding (years)**				
≤2	38 (33.0)	533 (22.6)	1.0 (reference)	1.0 (reference)
3–4	27 (23.5)	579 (24.5)	0.65 (0.39–1.09)	0.61 (0.36–1.01)
5–6	24 (20.9)	481 (20.4)	0.70 (0.41–1.18)	0.61 (0.36–1.06)
≤7	26 (22.6)	767 (32.5)	**0.48 (0.29–0.79)** **	**0.40 (0.23–0.70)** **
**Use of hormonal contraceptives** ^d^				
Yes	37 (30.8)	838 (32.8)	0.91 (0.61–1.36)	0.87 (0.58–1.30)
No	83 (69.2)	1717 (67.2)	1.0 (reference)	1.0 (reference)
**Menopausal status**				
Premenopausal	94 (71.8)	1858 (70.0)	1.0 (reference)	1.0 (reference)
Postmenopausal	37 (28.2)	795 (30.0)	0.92 (0.62–1.36)	0.91 (0.50–1.67)
**Family history of breast cancer**				
Yes	11 (8.7)	103 (4.1)	**2.25 (1.17–4.30)** *	**2.22 (1.16–4.26)** *
No	115 (91.3)	2418 (95.9)	1.0 (reference)	1.0 (reference)
**Irregular menses**				
Yes	5 (4.90)	88 (4.7)	1.04 (0.41–2.63)	1.05 (0.42–2.66)
No	97 (95.1)	1782 (95.3)	1.0 (reference)	1.0 (reference)

Abbreviations: CI, confidence interval; SD, standard deviation; BMI, body mass index.

a The age at the time of interview for controls.

b Underweight BMI: <18.5 kg/m^2^; healthy weight BMI: 18.5-<25 kg/m^2^; overweight i BMI: 25.0-<30.0 kg/m^2^; and obese BMI: ≥30 kg/m^2^.

c At least one alcoholic beverage a week for one year or longer.

d Hormonal contraceptives include oral, injectable, implant, and intrauterine contraceptive devices.

e Adjustment for age category and menopausal status.

* *P* < 0.05

** *P* < 0.01

*** *P* < 0.001.

**Table 4:** Multivariable logistic regression of the association between benign breast disease and breast cancer, overall and by country

		Model 1	Model 2	Model 3
	Crude OR (95% CI)	aOR^a^ (95% CI)	aOR^b^ (95% CI)	aOR^c^ (95% CI)
**“Yes” Self-reported BBD** *(reference: “No”***)**				
Overall	**1.43 (1.14–1.78)** **	**1.47 (1.17–1.86)** **	**1.39 (1.08–1.78)** *	**1.47 (1.13–1.91)** **
Nigeria	1.19 (0.93–1.52)	1.19 (0.92–1.55)	1.22 (0.92–1.60)	1.29 (0.97–1.72)t
Cameroon	**2.64 (1.36–5.14)** **	**2.74 (1.38–5.43)** **	**2.16 (1.05–4.42)** *	**2.21 (1.00–4.91)** *
Uganda	**4.27 (1.71–10.66)** **	**4.28 (1.66–11.06)** **	**5.49 (1.44–21.00)** *	**14.70 (2.70–79.94)** **
**“Yes” Biopsied BBD** *(reference: “No”***)**				
Overall	**2.61 (1.68–4.04)** ***	**3.03 (1.90–4.83)** ***	**2.70 (1.63–4.48)** ***	**3.11 (1.78–5.44)** ***

Abbreviations: OR, odds ratio; AOR, adjusted odds ration; CI, confidence interval; BBD, benign breast disease.

a Adjusted for age, level of education, study site (only for all-country model), ethnicity, and menopausal status.

b Adjusted for all covariates in model 1, total breastfeeding duration, parity, and age at first live birth.

c Adjusted for all covariates in models 1 and 2, height, waist-hip-ratio, and family history of breast cancer

* *P* < 0.05

** *P* < 0.01

*** *P* < 0.001.

† *P* = 0.082

**Table 5:** Mediation analysis of benign breast disease in the associations between selected risk factors and breast cancer

	Natural Direct Effect	Natural Indirect Effect	Total Effect	Proportion Mediated (%)
	AOR (95% CI)	AOR (95% CI)	AOR (95% CI)	
**BMI (kg/m^2^)**	**0.974 (0.963–0.984)** *	0.999 (0.997–1.002)	**0.973 (0.962–0.984)** *	1.9
**Alcohol consumption** ^a^	**1.491 (1.192–1.866)** ***	1.011 (0.986–1.037)	**1.508 (1.204–1.889)** *	3.3
**Age at menarche (years)**	**0.968 (0.940–0.997)** *	0.996 (0.985–1.008)	**0.965 (0.934–0.996)** *	9.8
**Age at first live birth (years)**	**1.018 (1.004–1.032)** *	1.000 (0.999–1.002)	**1.018 (1.004–1.032)** *	0.6
**Mean Duration of breastfeeding per live birth (months)**	**0.960 (0.949–0.970)** ***	0.999 (0.995–1.002)	**0.958 (0.947–0.969)** ***	8.0
**Use of hormonal contraceptives** ^b^	**0.803 (0.705–0.915)** **	1.000 (0.981–1.019)	**0.803 (0.703–0.916)** **	0.1
**Family history of BC**	**1.539 (1.165–2.032)** **	1.014 (0.987–1.042)	**1.561 (1.181–2.063)** **	4.0

Abbreviations: AOR, adjusted odd ratio; CI, confidence interval; cm, centimeter; BMI, body mass index.

a At least one alcoholic beverage a week for one year or longer.

b Hormonal contraceptives included oral, injectable, implant, and intrauterine contraceptive device.

c Defined as having at least one alcoholic beverage a week for the past 6 months or longer.

* *P* < 0.05

** *P* < 0.01

*** *P* < 0.001.

## Data Availability

The datasets analyzed during this study are not publicly available due to concerns about patient privacy, in particular, the breast cancer cases recruited from the four hospitals involved in the study.
